# Supplementary material for: Molecular basis of differential HLA class I-restricted T cell recognition of a highly networked HIV peptide
Source: Nat Commun. 2023 May 22;14:2929. doi: 10.1038/s41467-023-38573-8 (PMC10202051; doi:10.1038/s41467-023-38573-8)
Supplement: Supplementary file 1 — Supplementary Information [file 41467_2023_38573_MOESM1_ESM.pdf]

Supplementary Information

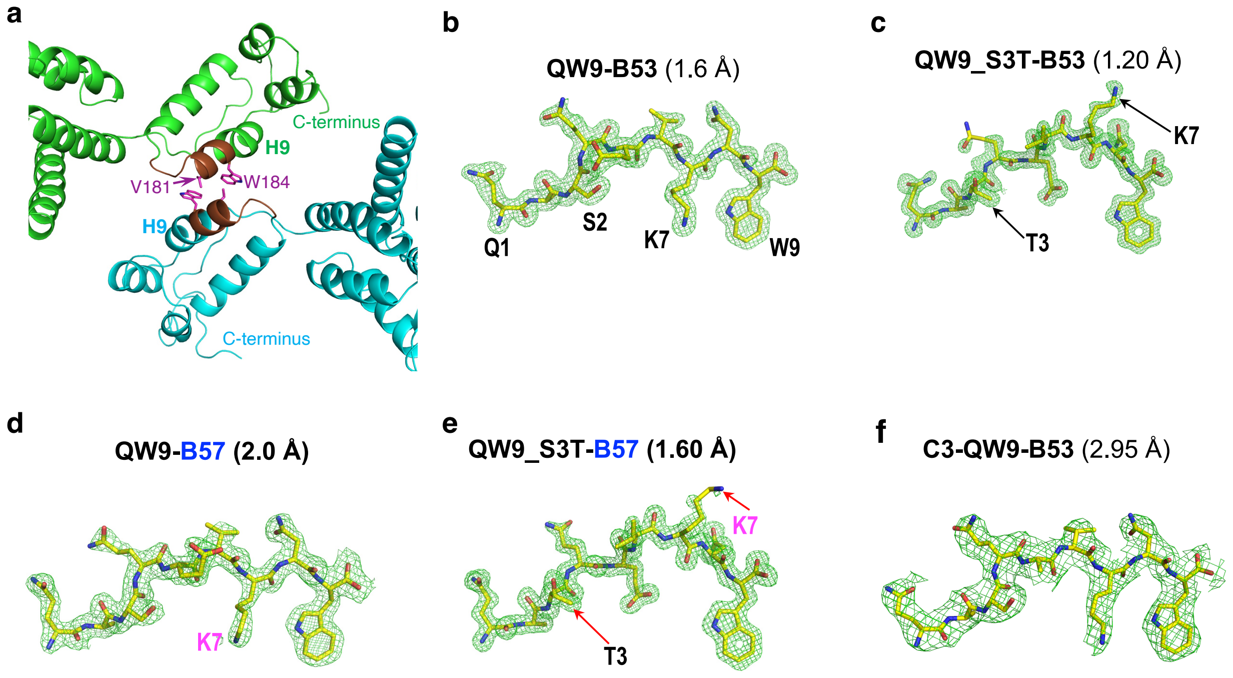

Supplementary Figure 1. Highly networked epitope QW9 presented by B53 and B57

a, The location of QW9 (colored in purple) in the assembled HIV-1 capsid protein Gag p24 (PDB: 3J4F). Two p24 protomers packed along a pseudo-dyad between two hexamers are shown in green and cyan ribbons, respectively. Residues $V6^{QW9}$ and $W9^{QW9}$ located at the p24 H9 helix are colored in purple. **b**-**f**, The electron density maps of QW9 and QW9_S3T in B53, B57 and C3-QW9-B53 structures. The map is shown as green mesh (2mFo-DFc, contoured at 1.2σ).

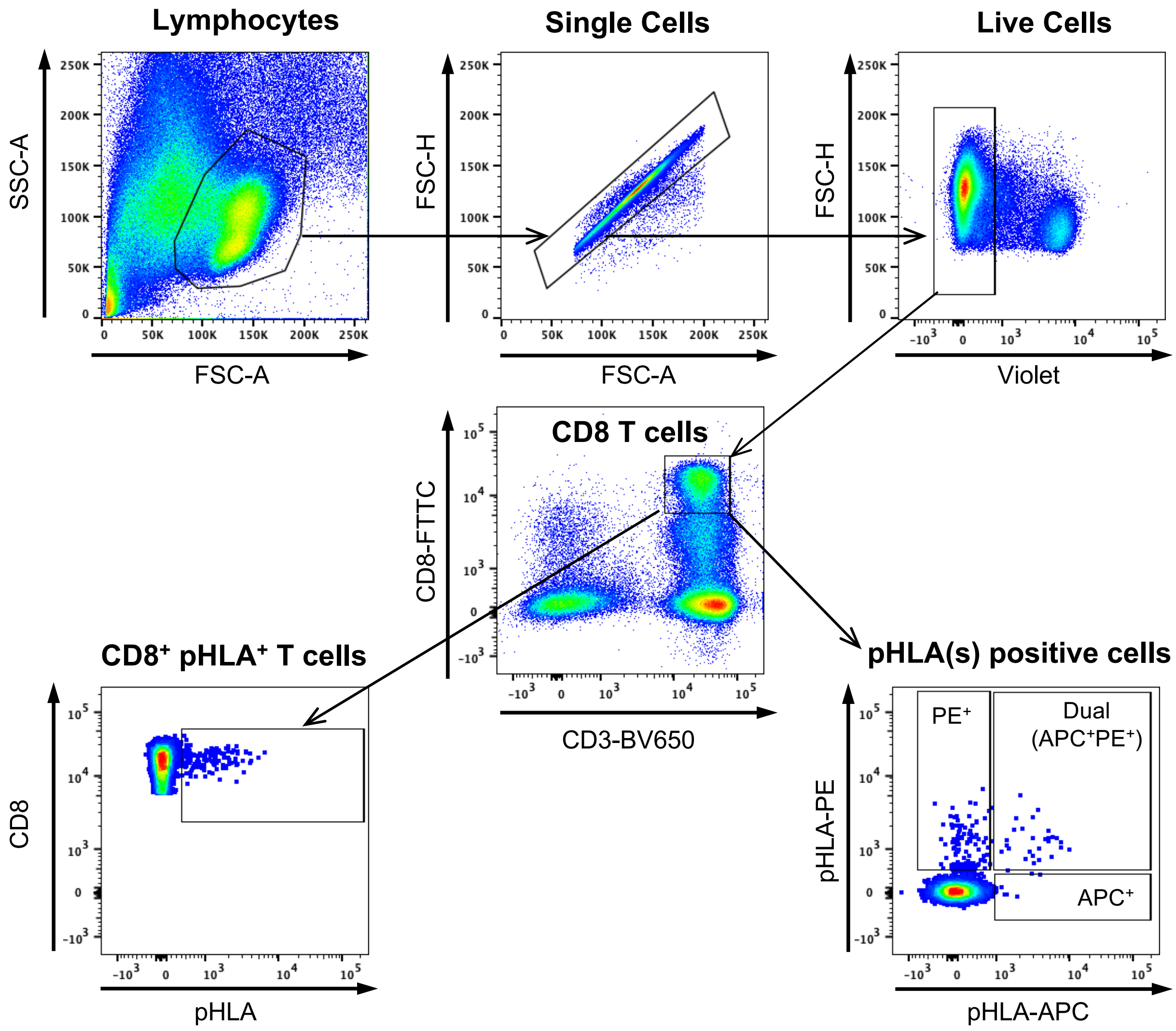

Supplementary Figure 2. Flow cytometry gating strategy for tetramer staining assays

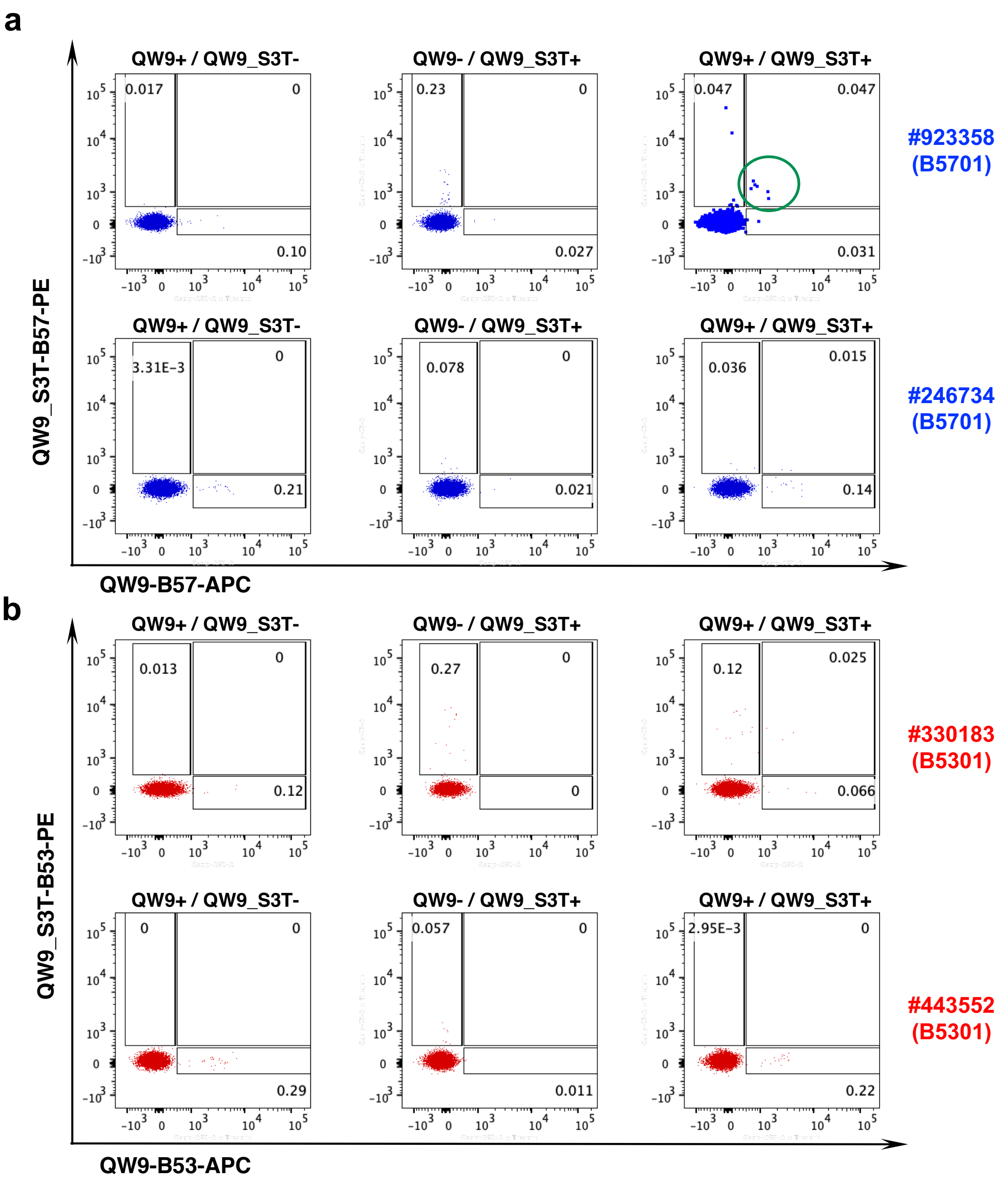

Supplementary Figure 3. Tetramer staining for additional B57⁺ and B53⁺ individuals

a, b, Representatives of tetramer-specific CTLs quantitation for B57⁺ (blue) and B53⁺ (red) individuals by flow cytometry (duplicate number: n=3). Columns left to right show QW9 tetramer, QW9_S3T tetramer, and dual tetramer staining. Rows depict individual subjects expressing B57 or B53, as indicated. Convincing dual QW9+/QW9_S3T+ tetramer staining population from a B57⁺ individual is highlighted in the green circle.

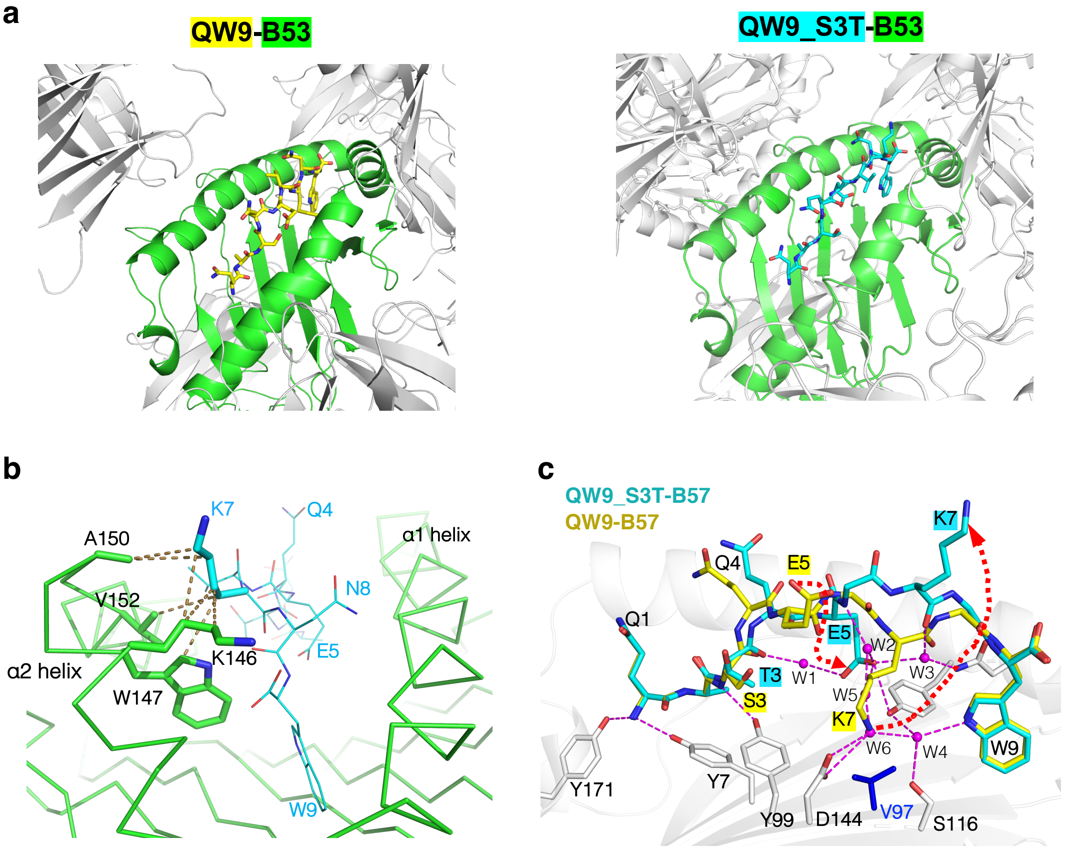

Supplementary Figure 4. Structural features of QW9 and QW9_S3T complexed with B53

a, The protein packing in the crystals of QW9-B53 (left panel) and QW9_S3T-B53 (right panel). The green ribbon in each panel is a representative of the QW9-B53 or QW9_S3T-B53 molecule in the respective crystal, and grey ribbons are the same protein molecules packing around the antigen binding region of a representative pHLA. Both peptides have no contact with their adjacent molecules. **b**, The stabilizing contacts between the sidechain of $K7^{QW9\_S3T}$ (cyan stick) and B53 (green skeleton). The hydrophobic contacts between $K7^{QW9\_S3T}$ and B53 are drawn with orange broken lines. **c**, The overlay of $QW9\_S3T^{B57}$ (cyan stick) onto $QW9^{B57}$ (yellow stick) in the context of the same B57 (white ribbon). Only key hydrogen bonds (magenta broken lines) and water molecules (magenta spheres) involved in the interaction between QW9_S3T and B57 are shown.

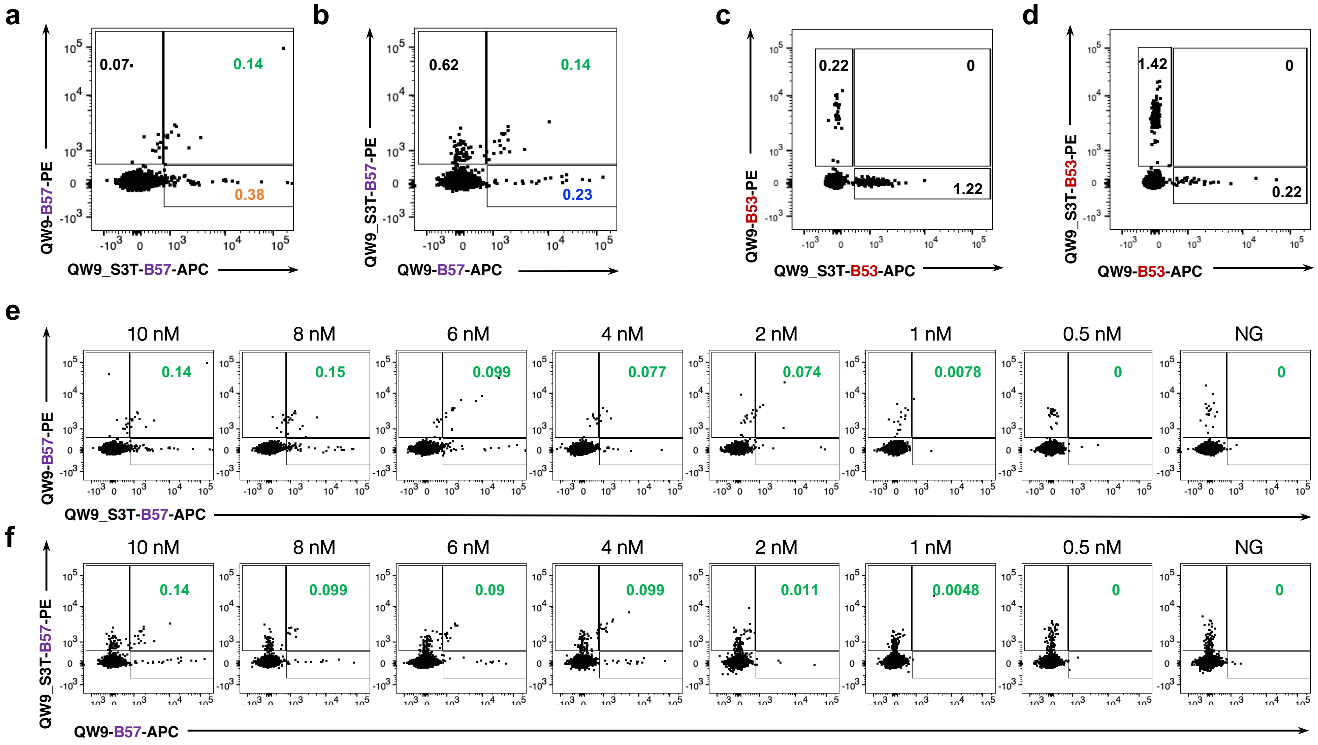

Supplementary Figure 5. pHLA tetramer titration for comparing cross-reactive TCR binding with QW9-B57 vs. QW9_S3T-B57

a-d, B57-restricted (**a-b**) or B53-restricted (**c-d**) QW9 responders were stained with fixed concentrations of PE-conjugated pHLA tetramer and titrated concentrations of APC-conjugated pHLA tetramers, as indicated by plot axes. Representative flow plots from the highest staining concentration are shown. **e**, B57^{+} PBMCs were stained with fixed concentration (10 nM) of PE-conjugated QW9-B5701 tetramer and titrated concentrations of APC-conjugated QW9_S3T-B57 tetramers, as indicated by X-axis. **f**, B57^{+} PBMCs were stained with fixed concentration (10 nM) of PE-conjugated QW9_S3T-B57 tetramer and titrated concentrations of APC-conjugated QW9-B57 tetramers, as indicated by X-axis. The duplicate number for each experiment, n=3.

Supplementary Table 1. Network scores of the individual amino acids of QW9 in gag p24

p24 gag (QW9)	Network Score
GLN176 (Q1)	2.110361193
ALA177 (A2)	3.797598535
SER178 (S3)	-0.950558885
GLN179 (Q4)	-1.146693637
GLU180 (E5)	-1.052420466
VAL181 (V6)	2.028056393
LYS182 (K7)	3.353713836
ASN183 (N8)	-1.068618976
TRP184 (W9)	2.270120659

Supplementary Table 2. Characteristics of the research participants for functional examination

	Participants ID	Yrs Dx to Sample Collection	Age at Sample Collection	Viral Load (RNA copies/ml)	CD4 count (cells/uL)	Gender	Race	HIV Risk	HLA
B*5701	**164007**	23	52	<75	1437	Female	AA/Hispanic	IVDU	A*0101, 0301; B*4102, 5701; Cw*0602, 1202
	**970489**	5	30	493	552	Male	Caucasian	MSM	A*0201, 3201; B*4001, 5701,; Cw*0501, 0602
	**900837**	22	49	355	664	Male	Caucasian	MSM	A*1101, 1101; B*1402, 5701; Cw*0602, 0802
	**542735**	13	43	<75	668	Male	Unknown	MSM	A*0201, 3001; B*5301, 5701; Cw*0413, 0602
	**264095**	1	52	184	682	Male	Caucasian	MSM	A*0301, 0301; B*1402, 5701; Cw*0602, 0801
	**246734**	2	65	<20	609	Male	Unknown	Unknown	A*0301, 3101; B*2705, 5701; Cw*0127, 0602
	**534694**	7	59	5450	663	Male	Unknown	Unknown	A*0201, 0201; B*2705, 5701; Cw*0102, 0602
	**923358**	6	62	<20	893	Male	White	MSM	A*0101, 0301; B*0702, 5701; Cw*0602, 0702
B*5301	**912103**	9	37	552	493	Male	White/Hispanic	MSM	A*3001, 3601; B*4402, 5301; Cw*0401, 0501
	**443552**	23	54	48	849	Male	AA	IVDU	A*0201, 0201; B*4901, 5301; Cw*0401, 0701
	**187948**	1	23	<50	1157	Female	Hispanic	Heterosexual	A*6801, 74; B*5301, 5701; Cw*0401, 0602
	**423060**	9	50	439	706	Male	AA	MSM	A*2301, 2902; B*3102, 5301; Cw*0602, 0602
	**616147**	6	40	400	772	Male	Caucasian	MSM	A*2301, 6801; B*1801, 5301; Cw*0401, 0701
	**973241**	9	50	2940	266	Male	Unknown	Unknown	A*3002, 3303; B*4403, 5301; Cw*0401, 0602
	**330183**	11	44	3180	1038	Male	Unknown	Unknown	A*0201, 6802; B*0702, 5301; Cw*0401, 0702

Supplementary Table 3. Diffraction data and refinement statistics

Protein (PDB code)	QW9-B53 (7R7V)	QW9_S3T-B53 (7R7W)	QW9-B57 (7R7X)	QW9_S3T-B57 (7R7Y)	C3 TCR (7R7Z)	C3 TCR-QW9-B53 (7R80)
**Data collection**						
Space group	$P\ 2_1\ 2_1\ 2_1$	$P\ 2_1\ 2_1\ 2_1$	$P\ 2_1\ 2_1\ 2_1$	$P\ 2_1\ 2_1\ 2_1$	$P\ 1\ 2_1\ 1$	$P\ 2_1\ 2_1\ 2_1$
Cell dimensions						
a, b, c (Å)	50.6 82.0 109.8	50.7 82.4 110.4	69.5 82.6 157.3	50.3 81.8 109.9	43.2 59.4 82.7	60.31 64.814 220.9
α, β, γ (°)	90 90 90	90 90 90	90 90 90	90 90 90	90 90.5 90	90 90 90
Resolution (Å)	1.60	1.17	2.10	1.60	2.29	2.95
R_{merge} (%)	11.6 (110.1)	4.8 (86.6)	6.8 (63.8)	9.5 (65.1)	11.1 (90.4)	13.8 (129.9)
I / σ	21.4 (1.7)	25.5 (1.2)	17.7 (2.4)	61.7 (7.9)	12.0 (1.5)	14.0 (1.3)
Completeness (%)	99.1 (99.8)	97.6 (95.6)	99.9 (99.3)	100 (100)	98.1 (90.2)	90.2 (90.3)
Redundancy	5.5(5.6)	5.0(4.2)	6.3 (6.0)	18.5 (18.5)	5.1(2.6)	4.4 (4.0)
**Refinement**						
Resolution (Å)	50.00 -1.60 (1.63-1.60)	50.00 - 1.17 (1.19 - 1.17)	39.94 - 2.10 (2.17 - 2.10)	32.82 - 1.60 (1.66 - 1.60)	41.34 - 2.29 (2.37 -2.29)	50.00 - 2.95 (3.00 - 2.95)
No. reflections	60504 (6032)	151816 (14712)	53661 (5270)	60429 (5868)	17276 (899)	17414 (1279)
R_{work} / R_{free}	17.4 (22.7) / 19.8 (24.2)	13.2 (25.5) / 15.7 (27.3)	18.4 (23.2) / 21.9 (27.9)	15.3 (18.3) / 18.9 (23.6)	21.5 (28.3) / 25.2 (33.7)	23.2 (30.1) / 28.6 (35.9)
No. atoms	3618	3900	6915	3779	3552	6407
Protein	3205	3268	6326	3191	3447	6258
Ligand/ion	12	None	16	18	22	None
Water	401	632	573	570	83	149
B-factors	30.42	22.30	34.3	20.81	40.62	94.58
Protein	29.14	18.95	33.64	18.18	40.79	95.13
Ligand/ion	45.18	None	71.11	51.3	36.99	None
Water	40.24	39.59	40.59	34.53	34.65	71.25
R.m.s. deviations						
Bond lengths (Å)	0.015	0.012	0.004	0.009	0.002	0.007
Bond angles (°)	1.37	1.29	0.66	1.07	0.54	1.1

*Number of xtals for each structure should be noted in footnote. *Values in parentheses are for highest-resolution shell.

Supplementary Table 4. Buried surface area (BSA) contributed from each component of the C3-QW9-B53 complex

	Loop	BSA (Å^2)	Contribution	Contribution (Sum)
**TCR α**	CDR1α	168.8	17.1%	38.7%
	CDR2α	0.8	0	
	CDR3α	213.2	21.6%	
**TCR β**	CDR1β	100.0	10.1%	61.3%
	CDR2β	174.2	17.6%	
	CDR3β	330.7	33.6%	
**MHC**		746.2		75.6%
**QW9**		240.8		24.4%
**Total**		1974.7		

Supplementary Table 5. Least-squares superpositions of apo B53 and C3 onto C3-QW9-B53 complex

r.m.s.d (Å)	B*5301	C3 TCR
α1α2	0.48(0.88)	
QW9	0.19(0.45)	
Vα		0.76(1.35)
Vβ		0.49(1.12)
CDR1α		1.22(2.15)
CDR2α		0.26(1.26)
CDR3α		0.38(0.59)
CDR1β		0.28(1.55)
CDR2β		0.18(0.33)
CDR3β		0.68(1.94)
