# Supplementary material for: Molecular basis of differential HLA class I-restricted T cell recognition of a highly networked HIV peptide
Source: Nat Commun. 2023 May 22;14:2929. doi: 10.1038/s41467-023-38573-8 (PMC10202051; doi:10.1038/s41467-023-38573-8)
Supplement: Supplementary file 3 — Reporting Summary [file 41467_2023_38573_MOESM3_ESM.pdf]

nature portfolio

Corresponding author(s): Xiaolong Li; Bruce D. Walker; Jia-huai Wang

Last updated by author(s): 03/17/2023

Reporting Summary

Nature Portfolio wishes to improve the reproducibility of the work that we publish. This form provides structure for consistency and transparency in reporting. For further information on Nature Portfolio policies, see our Editorial Policies and the Editorial Policy Checklist.

Statistics

For all statistical analyses, confirm that the following items are present in the figure legend, table legend, main text, or Methods section.

n/a	Confirmed	
☐	☒	The exact sample size (*n*) for each experimental group/condition, given as a discrete number and unit of measurement
☐	☒	A statement on whether measurements were taken from distinct samples or whether the same sample was measured repeatedly
☐	☒	The statistical test(s) used AND whether they are one- or two-sided<br>*Only common tests should be described solely by name; describe more complex techniques in the Methods section.*
☒	☐	A description of all covariates tested
☒	☐	A description of any assumptions or corrections, such as tests of normality and adjustment for multiple comparisons
☐	☒	A full description of the statistical parameters including central tendency (e.g. means) or other basic estimates (e.g. regression coefficient) AND variation (e.g. standard deviation) or associated estimates of uncertainty (e.g. confidence intervals)
☐	☒	For null hypothesis testing, the test statistic (e.g. *F*, *t*, *r*) with confidence intervals, effect sizes, degrees of freedom and *P* value noted<br>*Give P values as exact values whenever suitable.*
☒	☐	For Bayesian analysis, information on the choice of priors and Markov chain Monte Carlo settings
☒	☐	For hierarchical and complex designs, identification of the appropriate level for tests and full reporting of outcomes
☒	☐	Estimates of effect sizes (e.g. Cohen's *d*, Pearson's *r*), indicating how they were calculated

Our web collection on statistics for biologists contains articles on many of the points above.

Software and code

Policy information about availability of computer code

Data collection	SBCCOLLECT(for X-ray diffraction data collection) ; FACSAria II (BD Biosciences); LSR Fortessa (BD biosciences); BiaEvaluation (cytiva); MiSeq Control Software(Illumina 4.0.0.1769)
Data analysis	HKL3000 (for X-ray data index, integrate and scale); CCP4i 7.1 (for data format conversion and analysis); Phenix-1.18.2 (for structure determination and refinement); COOT-0.8.9.2 (for structure model build and check); PyMol 2.4.1(for structural analysis); UNICORN 7 (for Protein purification); OriginPro2017 (for curve figure); IMGT v-quest (for identification of TCR gene segments and CDR3 regions); Flowjo (cell subsets analysis); Prism 7/8 (for statistic analysis); BiaEval software (for kinetic parameters calculation); MIxCR version 3.0.13 (for scTCR sequencing analysis).

For manuscripts utilizing custom algorithms or software that are central to the research but not yet described in published literature, software must be made available to editors and reviewers. We strongly encourage code deposition in a community repository (e.g. GitHub). See the Nature Portfolio guidelines for submitting code & software for further information.

Data

Policy information about availability of data

All manuscripts must include a data availability statement. This statement should provide the following information, where applicable:

- Accession codes, unique identifiers, or web links for publicly available datasets
- A description of any restrictions on data availability
- For clinical datasets or third party data, please ensure that the statement adheres to our policy

The data that support this study are available from the corresponding authors upon request. All structural data have been deposited in the Protein Data Bank (https://www.rcsb.org), with PDB codes: 7R7V (https://www.rcsb.org/structure/7R7V), 7R7W (https://www.rcsb.org/structure/7R7W), 7R7X (https://www.rcsb.org/structure/7R7X), 7R7Y (https://www.rcsb.org/structure/7R7Y), 7R7Z (https://www.rcsb.org/structure/7R7Z), 7R80 (https://www.rcsb.org/structure/7R80).
The source data underlying Figs. 1d-g, 2g, 4b, 4d-f, 6i, 7a, b, f are provided in the Source Data file. The Source Data are provided with this paper.

Human research participants

Policy information about studies involving human research participants and Sex and Gender in Research.

Reporting on sex and gender	Our study is not related to gender.
Population characteristics	Characteristics of the research subjects are shown in Extended Data Table 1. Elite controllers were defined as having plasma HIV-1 RNA below the level of detection for the ultrasensitive assay (< 75 RNA copies/mL by cDNA or < 50 copies/mL by ultrasensitive PCR) without antiretroviral therapy and viremic controllers as having HIV-1 RNA < 2000 RNA copies/mL without antiretroviral therapy.
Recruitment	Our study has no recruitment.
Ethics oversight	All samples were obtained from cryopreserved PBMC from HIV-1-infected individuals according to protocols approved by the Institutional Review Board of the Massachusetts General Hospital.

Note that full information on the approval of the study protocol must also be provided in the manuscript.

Field-specific reporting

Please select the one below that is the best fit for your research. If you are not sure, read the appropriate sections before making your selection.

☒ Life sciences ☐ Behavioural & social sciences ☐ Ecological, evolutionary & environmental sciences

For a reference copy of the document with all sections, see nature.com/documents/nr-reporting-summary-flat.pdf

Life sciences study design

All studies must disclose on these points even when the disclosure is negative.

Sample size	over 70 Cryopreserved PBMC samples from 14 HIV-1-infected individuals. They were divided into two groups based on their HLA genotypes. For biological comparative study, each group includes over 5 different individuals. For PBMC vials, it depend on the target cell numbers for each experiment and the numbers of duplicates.
Data exclusions	No data was excluded.
Replication	For cell analyses, results are reported from 3 experiments; For ex vivo INF-γ Elispot assay results are reported from 3 technical duplicates experiment, measurements; For protein-protein binding measurements, results are reported from 2 experiments; For protein thermal melt measurement, results are reported from 3 experiments. All attempts to the biological and biophysical measurements were successful and included in the data analysis. All experiments did not require measurements at multiple time points.
Randomization	As no interventions were included in this study, randomization of participants was not performed.
Blinding	All studies were performed in vitro. Blinding was not performed for this study as the knowledge of patient HIV status was required for selection of samples for HIV-elimination and recognition assays.

Reporting for specific materials, systems and methods

We require information from authors about some types of materials, experimental systems and methods used in many studies. Here, indicate whether each material, system or method listed is relevant to your study. If you are not sure if a list item applies to your research, read the appropriate section before selecting a response.

Materials & experimental systems

n/a	Involved in the study
☐	☒ Antibodies
☐	☒ Eukaryotic cell lines
☒	☐ Palaeontology and archaeology
☒	☐ Animals and other organisms
☒	☐ Clinical data
☒	☐ Dual use research of concern

Methods

n/a	Involved in the study
☒	☐ ChIP-seq
☐	☒ Flow cytometry
☒	☐ MRI-based neuroimaging

Antibodies

Antibodies used

Anti-human CD3 BV650 (BD Biosciences, UCHT1, Cat#300468); Anti-human CD8 FITC (Biolegend, RPA-T8, Cat#301006); Human IFN-gamma ELISpotBASIC kit (ALP) (Cat# 3420-2A, Mabtech)

Validation

Anti-human CD3 BV650 (BD Biosciences, UCHT1, Cat#300468)
Isotype: Mouse IgG1, κ
Reactivity: Human, Cross-Reactivity: Chimpanzee
Antibody Type: Monoclonal
Application: FC - Quality tested
Application Notes: Additional reported applications (for the relevant formats) include: immunohistochemical staining of acetone-fixed frozen sections4,6,7 and formalin-fixed paraffin-embedded sections11, immunoprecipitation1, activation of T cells2,3,5, Western blotting9, and spatial biology (IBEX)16,17. The LEAF™ purified antibody (Endotoxin < 0.1 EU/µg, Azide-Free, 0.2 µm filtered) is recommended for functional assays (Cat. No. 300413, 300414, and 300432). For highly sensitive assays, we recommend Ultra-LEAF™ purified antibody (Cat. No. 300437, 300438, 300465, 300466, 300473, 300474) with a lower endotoxin limit than standard LEAF™ purified antibodies (Endotoxin < 0.01
Application References:
Salmeron A, et al. 1991. J. Immunol. 147:3047. (IP)
Graves J, et al. 1991. J. Immunol. 146:2102. (Activ)
Lafont V, et al. 2000. J. Biol. Chem. 275:19282. (Activ)
Thakral D, et al. 2008. J. Immunol. 180:7431. (FC)
Van Dongen JJM, et al. 1988. Blood 71:603. (WB)
Yoshino N, et al. 2000. Exp. Anim. (Tokyo) 49:97. (FC)
Pollard, K. et al. 1987. J. Histochem. Cytochem. 35:1329. (IHC)
Luckashenak N, et al. 2013. J. Immunol. 190:27.

Anti-human CD8 FITC (Biolegend, RPA-T8, Cat#301006)
Isotype: Mouse IgG1, κ
lReactivity: Human, Cross-Reactivity: Chimpanzee, Baboon, Cynomolgus, Rhesus, Pigtailed Macaque, Sooty Mangabey
Antibody Type: Monoclonal
Application: FC - Quality tested
Application Notes: The RPA-T8 antibody does not block the binding of HIT8a antibody to CD8a. Additional reported applications of this antibody (for the relevant formats) include: immunohistochemical staining of paraformaldehyde-fixed frozen sections3 and costimulation of T cell responses4. This clone was tested in-house and does not work on formalin fixed paraffin-embedded (FFPE) tissue. The Ultra-LEAF™ purified antibody (Endotoxin <0.1 EU/µg, Azide-Free, 0.2 µm filtered) is recommended for functional assays (Cat. Nos. 301073 & 301074).
Application References:
Knapp W, et al. Eds. 1989. Leucocyte Typing IV. Oxford University Press. New York.
Schlossman S, et al. Eds. 1995. Leucocyte Typing V. Oxford University Press. New York.
Mack CL, et al. 2004. Pediatr. Res. 56:79. (IHC)
Magidovich E, et al. 2007. P. Natl. Acad. Sci. USA 104:13022.
Kmieciak M, et al. 2009. J. Transl. Med. 7:89. (FC)
Thakral D, et al. 2008. J. Immunol. 180:7431. (FC)
Yoshino N, et al. 2000. Exp. Anim. (Tokyo) 49:97. (FC)
Stoeckius M, et al. 2017. Nat. Methods. 14:865. (PG)

Human IFN-gamma ELISpotBASIC kit (ALP) (Cat# 3420-2A, Mabtech)
Application: ELISpot
REACTIVITY: Human
CONTENTS: Capture mAb (1-D1K); Biotinylated detection mAb (7-B6-1); Streptavidin-ALP
References:
Currier, J. R., et al. (2002). A panel of MHC class I restricted viral peptides for use as a quality control for vaccine trial ELISPOT assays. Journal of immunological methods.
Czerkinsky, C., et al. (1988). Reverse ELISPOT assay for clonal analysis of cytokine production. I. Enumeration of gamma-interferon-secreting cells. Journal of immunological methods.
Gubin, M. M., et al. (2014). Checkpoint blockade cancer immunotherapy targets tumour-specific mutant antigens. Nature.

Ott, P. A., et al. (2017). An immunogenic personal neoantigen vaccine for patients with melanoma. Nature.
Sekine, T., et al. (2020). Robust T Cell Immunity in Convalescent Individuals with Asymptomatic or Mild COVID-19. Cell.

CD3ε Recombinant monoclonal antibody (12F6) (Mouse IgG2aκ) (Cat# ENZ-ABS621-0200, Enzo Life Sciences)
Alternative Name: T-cell surface glycoprotein CD3 ε chain, T-cell surface antigen T3/Leu-4 epsilon chain, CD3e
Clone: 12F6
Host: Mouse
Isotype: IgG2aκ
Immunogen: Human CD3.
UniProt ID: P07766
Species reactivity: Human
Applications: Flow Cytometry
Purity Detail: Protein A affinity purified.
Formulation: Liquid. In PBS containing 0.02% Proclin 300.
Use/Stability: Stable at 4°C for up to 3 months. For longer storage, aliquot and store at -20°C.
Handling: Avoid freeze/thaw cycles.
Shipping: Shipped on Blue Ice
Short Term Storage: +4°C
Long Term Storage: -20°C

Eukaryotic cell lines

Policy information about cell lines and Sex and Gender in Research

Cell line source(s)	HEK293T17 cells were obtained from ATCC (Catalogue number CRL-11268); Jurkat, Clone E6-1 were obtained from ATCC (TIB-152™);<br>Autologous Epstein-Barr virus-transformed B cell lines were used as targets in the chromium release assays. Ten million frozen PBMC were thawed and resuspended in 1mL of RMPI, 1.5mL of fetal bovine serum and 1.5mL of unconcentrated supernatant of Epstein-Barr virus. Cyclosporine A was added in a 1μg/mL concentration. Cells were cultured for 6 to 8 weeks at 37°C and 5% CO_2.
Authentication	Cell lines were authenticated by STR profiling.
Mycoplasma contamination	Cell lines were not tested for mycoplasma.
Commonly misidentified lines (See ICLAC register)	The original cell line was obtained from ATCC. This specific clone of this cell line is optimal for retrovirus production. No commonly misidentified cell lines were used.

Flow Cytometry

Plots

Confirm that:

- [x] The axis labels state the marker and fluorochrome used (e.g. CD4-FITC).
- [x] The axis scales are clearly visible. Include numbers along axes only for bottom left plot of group (a 'group' is an analysis of identical markers).
- [x] All plots are contour plots with outliers or pseudocolor plots.
- [x] A numerical value for number of cells or percentage (with statistics) is provided.

Methodology

Sample preparation

Tetramer staining
Biotinylated HLA-B*5701 and HLA-B*5301 monomers refolded with QW9 (QASQEVKNW) or the mutant peptide QW9_S3T (QATQEVKNW) were customed from the company immunAware. Monomers were then tetramerized using phycoerythrin (PE)- or allophycocyanin (APC)-conjugated streptavidin (Cat. 405203; Cat. 405207, Biolegend). Tetramers were validated to rule out non-specific binding with HLA-matched HIV-negative samples. Cryopreserved PBMCs from research participants were incubated with the corresponding QW9 and/or mutant tetramers for 25 minutes at 37°C and 5% CO_2. The cells were then stained by LIVE/DEAD™ Fixable Violet dye (Cat. L34964, ThermoFisher) for viability (1/1000 (v/v) dilution) and anti-CD3 (Cat. 300468, BioLegend) and anti-CD8 (Cat. 301006, BioLegend) antibodies (1/100 (v/v) dilution) for surface markers and 5nM corresponding tetramer for TCR.

Proliferation assay
PBMCs from the study participants were stained with carboxyfluorescein succinimidyl ester (CFSE, Cat. C34554, ThermoFisher) by incubating the cells with 1μM CFSE solution (1/1000 (v/v) dilution) for 20 minutes at 37°C and 5% CO_2 and then washed twice in R10 media. The cells were plated at 250,000 cells per well in a 96-well round-bottom plate with 0.5μg/mL of the corresponding peptides for seven days. The peptides (United Biosystems) used were QW9 (QASQEVKNW) and QW9_S3T (QATQEVKNW). The negative control well had no peptide, and the positive control well had PHA (Cat. 11249738001, Sigma) at 5μg/mL. On day 7, the cells were stained with IVE/DEAD™ Fixable Violet dye (Cat. L34964,

	ThermoFisher) for viability (1/1000(v/v) dilution), Anti-human CD3 BV650 (Cat. 300468, BioLegend) for CD3 (1/100(v/v) dilution), and Anti-human CD8 FITC (Cat.301006, BioLegend) for CD8 (1/100(v/v) dilution).
Instrument	All cell fluorescence analysis and sorting was performed on a BD FACS Aria II
Software	FlowJo, v7/v8 (BD)
Cell population abundance	Post-sort analysis was performed and confirmed within the defined gates (Extended Data Fig.2)
Gating strategy	See Fig.1, Extended Fig. 2, and the online method for full description. Briefly, At first, lymphocytes are gated using forward and side scatter. Subsequently, single cells are gated using forward scatter height and wide followed by side scatter height and wide. Live cells are gated using forward scatter area and Violet negative cells. Next, CD3+CD8+ cells are gated. Finally single HLA-tetramer+ or dual HLA-tetramer cells are gated.

☒ Tick this box to confirm that a figure exemplifying the gating strategy is provided in the Supplementary Information.
